# Does community-based health insurance protect women from financial catastrophe after cesarean section? A prospective study from a rural hospital in Rwanda

**DOI:** 10.1186/s12913-022-08101-3

**Published:** 2022-05-31

**Authors:** Rachel Koch, Theoneste Nkurunziza, Niclas Rudolfson, Jonathan Nkurunziza, Laban Bakorimana, Holly Irasubiza, Kristin Sonderman, Robert Riviello, Bethany L. Hedt-Gauthier, Mark Shrime, Fredrick Kateera

**Affiliations:** 1 Program in Global Surgery and Social Change, Harvard Medical School, Boston, USA; 2 University of Utah, Salt Lake City, USA; 3 Department for Sport and Health Sciences, Epidemiology, Technical University of Munich, Munich, Germany; 4 WHO Collaborating Centre for Surgery and Public Health, Lund University, Lund, Sweden; 5 Partners In Health/Inshuti Mu Buzima, Kigali, Rwanda; 6 Department of Global Health and Social Medicine, Harvard Medical School, Boston, USA; 7 Center for Global Surgery Evaluation, Boston, USA

**Keywords:** Developing countries, Economic crisis, Health care reform, Health financing, Maternity services, Policy evaluation

## Abstract

**Background:**

The implementation of community-based health insurance in (CBHI) in Rwanda has reduced out of pocket (OOP) spending for the > 79% of citizens who enroll in it but the effect for surgical patients is not well described. For all but the poorest citizens who are completely subsidized, the OOP (out of pocket) payment at time of service is 10%. However, 55.5% of the population is below the international poverty line meaning that even this copay can have a significant impact on a family’s financial health. The aim of this study was to estimate the burden of OOP payments for cesarean sections in the context of CBHI and determine if having it reduces catastrophic health expenditure (CHE).

**Methods:**

This study is nested in a larger randomized controlled trial of women undergoing cesarean section at a district hospital in Rwanda. Eligible patients were surveyed at discharge to quantify household income and routine monthly expenditures and direct and indirect spending related to the hospitalization. This was used in conjunction with hospital billing records to calculate the rate of catastrophic expenditure by insurance group.

**Results:**

About 94% of the 340 women met the World Bank definition of extreme poverty. Of the 330 (97.1%) with any type of health insurance, the majority (*n* = 310, 91.2%) have CBHI. The average OOP expenditure for a cesarean section and hospitalization was $9.36. The average cost adding transportation to the hospital was $19.29. 164 (48.2%) had to borrow money and 43 (12.7%) had to sell possessions. The hospital bill alone was a CHE for 5.3% of patients. However, when including transportation costs, 15.4% incurred a CHE and including lost wages, 22.6%.

**Conclusion:**

To ensure universal health coverage (UHC), essential surgical care must be affordable. Despite enrollment in universal health insurance, cesarean section still impoverishes households in rural Rwanda, the majority of whom already lie below the poverty line. Although CBHI protects against CHE from the cost of healthcare, when adding in the cost of transportation, lost wages and caregivers, cesarean section is still often a catastrophic financial event. Further innovation in financial risk protection is needed to provide equitable UHC.

**Supplementary Information:**

The online version contains supplementary material available at 10.1186/s12913-022-08101-3.

## Background

Each year, 81 million people are pushed into poverty due to costs associated with surgical care [1]. Ensuring that all people have access to quality healthcare services while protecting them from financial hardship related to paying for healthcare is a key tenet of universal health coverage (UHC) [2]. With the more recent inclusion of essential and emergency surgery – including caesarean section (c-section) – as components of UHC, this is an area that demands further understanding [1, 3]. According to the World Health Organization (WHO) [4], ensuring increased access to c-section is crucial to achieving decreased maternal and fetal mortality. However, c-section rates in sub-Saharan Africa (SSA) [5], though rising, remain lower than the 10–15% predicted to be associated with decreased maternal and fetal mortality [6].

The financial cost of surgical care is a core barrier to achieving target c-section rates in SSA. In Burkina Faso, where the c-section rate is just 1.8%, c-sections are five times more expensive than vaginal delivery and 10% of households were still paying off debt up to 4 years later [7]. Removing user costs for surgery increases rates of c-section but does not address the multitude of access issues related to poverty [5]. In Rwanda, 97% of women deliver in facilities rather than at home and 13% deliver via c-section [8], but the financial ramifications of requiring this hospital-based procedure is not known.

Over 80% of the Rwandan population live rurally [8] as of the 2015 census. Although only 38.2% live below the Rwandan national poverty line, 55% live in extreme poverty according to the World Bank standard, which is defined as living on less than $1.90 per day [9]. The government spends 7.5% of total Gross Domestic Product on healthcare, which translates to $170 per capita per year [10]. Since 1999, all Rwandans have had access to community-based health insurance (CBHI) – locally called *Mutuelles de Santé*. This scheme was devised to decrease poverty in the post-genocide period, and membership became compulsory by the national CBHI law in 2007 (though there are no direct penalties if citizens do not enroll) leading to high levels of enrollment [11] which have been celebrated internationally. An early study found that having CBHI was associated with a fourfold increase in the likelihood of a patient seeking modern medical care [12]. CBHI covers a basic package of services and drugs from authorized providers anywhere in the country as determined by the Ministry of Health and Rwanda Social Security Board. A referral or transfer from a health center is required to access a higher level of care such as a hospital for c-section.

Household contributions to CBHI are based on a 4-tier wealth system (*Ubudehe*), which classifies the population based on socioeconomic status and property. Rwandans are charged an annual premium plus a co-pay at the point of service provision, both adjusted by their socioeconomic status. Category 1 includes the poorest of the population and Category 4 includes the highest wealth quartile [13]. Those enrolled are eligible to receive the same services, and both pre and postnatal care are included for all categories. *Ubudehe* Category 1 patients do not pay any annual premium or copay [14]; rather, the government and other donors pay 2000 Rwandan Francs (RWF) (~$2.40) per household member per year for them to be insured. Category 2 and 3 patients pay a premium of 3000RWF (~$3.51) per household member annually and a 10% co-pay for all hospital medical services. Category 4 patients pay 7000RWF (~$8.19) per household member annually and cover 10% of hospital medical costs. As of January 2016, 79% of Rwandans were enrolled in CBHI [15]. In the Eastern province, 78.6% of residents have insurance. Of those, 97.7% have CBHI, 4.7% have government or military insurance and 0.2% have private insurance [8]. Those who are uninsured must pay hospital fees daily. The implementation of CBHI in Rwanda has reduced out-of-pocket (OOP) spending by patients, increased utilization of maternal healthcare and decreased catastrophic expenditure [14]. However, a follow up study indicated that these effects were more dramatic for wealthier patients than the poor and had less of a beneficial reduction for those seeking inpatient services [16].

One in ten women in rural Rwanda deliver via c-section [8], thus, a substantial portion of the population is at risk for significant financial burden despite heavy subsidies. Two metrics have been used to quantify the risk of financial hardship from surgical care: the rate of impoverishing expenditure, defined as expenditure that pushes a household below the poverty line, and the rate of catastrophic health expenditure (CHE), defined as spending greater than 10% of total annual household expenditure [17]. Because nonmedical costs such as transportation, food while in the hospital, and lost wages during the hospitalization contribute to financial hardships [18], these must be considered when determining the overall financial risk for patients. Recent systematic review found rates of CHE for patients in SSA undergoing c-section to be 56–67% [19]. Understanding CHE related to a specific pathology, in this case from c-section deliveries, can help governments better protect their citizens from financial disaster [20].

No patient should experience financial catastrophe seeking essential surgical care. While previous work in Rwanda has estimated the cost of c-section delivery at a rural district hospital to be approximately $339 [21], the risk of catastrophic expenditure due to c-section has yet to be studied. We hypothesized that given the poverty of the population, this expense, even when subsidized by the government, would be catastrophic for many households, particularly when considering the added nonmedical costs of hospitalization. Using descriptive methodology, we analyzed data collected from patient surveys and hospital billing data to assess the rates of CHE in our patient population. The goal of this study was to measure the economic burden associated with having a c-section at a district hospital in Rwanda and the financial risk protection conferred by CBHI.

## Methods

### Study setting

This study was conducted at Kirehe District Hospital (KDH) which is located in the Eastern province of Rwanda, an approximately three-hour drive from the capital, Kigali. KDH is a 226-bed hospital with a catchment area of 350,000, which is run by the Ministry of Health with addtional support from Partners in Health, a USA-based non-governmental organization (NGO). Kirehe district has sixteen health centers that refer patients to KDH when a higher level of care is needed.

Women in labor first seek care at their local health center and are then transferred to KDH if a higher level of obstetric care, such as a c-section, is indicated. Once at KDH, women are assessed by the on-call General Practitioner, a bachelors-level physician with some additional training on cesarean deliveries, or the Obstetrician-Gynecologist who performs a c-section if the physician determines it to be necessary. The cost of the procedure is not discussed preoperatively. On average, women who deliver by c-section are discharged on postoperative day (POD) 3. The maternity department keeps paper charts to record details of the medical care provided. The billing department maintains a separate electronic record for each patient and collects payment from hospitalized patients daily. Though informal payments for healthcare are common in some parts of Africa [22], this is not the case in the Rwandan healthcare context; none of our patients reported using anything other than the Rwandan currency to pay.

### Study design and population

This cross-sectional study was nested in a parent study on c-section-associated surgical site infections [23]. In the parent study, all women ≥18 years who underwent c-section at KDH between November 2017 and October 2018 were enrolled (*n* = 1116). For this unfunded sub-study, we included a subset of the study population: women enrolled over a 3-month period between April 21 and July 28, 2018 in order to obtain 300 patients. The parent study excluded patients from the Mahama refugee camp and patients residing outside of Kirehe District as their c-section referral and follow-up patterns as well as base income levels were considered different from the general population. From previous work done in the region, we know that the demographic of this district closely resembles that of the other rural districts in Rwanda.

### Data collection and questionnaire

As part of the parent study, trained data collectors interviewed consenting patients on POD 1 to collect demographic data and details on transportation (time and costs) to the hospital. At the time of discharge, usually on POD 3, data collectors extracted additional data on clinical course from the hospital chart.

For this study, data collectors interviewed enrolled patients who agreed to respond to additional financial questions at the time of discharge outside the maternity ward to maintain confidentiality. The questions (Additional file 1: Appendix 1) were adapted from the Program in Global Surgery and Social Change (PGSSC) National Surgical Obstetric Anesthesia Plan surgical indicator questionnaire which was originally developed, validated and piloted by a group working in Uganda [24] [25]. We added the following to the core questionnaire: estimates of monthly household income; routine monthly or annual expenditures for food and drink, transportation, livestock, housing, school fees, and healthcare; and whether the patient had to borrow money or sell possessions to pay for the current hospitalization. Non-monetary income such as agricultural harvest was converted into RWF using the local goods prices at the time. All study data were entered directly during the interview into REDCap software [26]on tablets. Data collectors used the hospital billing online record (OpenMRS) to verify the insurance type for patients and the total cost, categorized by type of expense, charged to each patient for the hospitalization. Data collected from this system were recorded in Excel, to allow data collectors to adjust for variability in the formatting of billing receipts, and the extracted data were then merged into a single dataset for analysis. Participants received no compensation for their participation in this or any part of the study.

### Key variables

We grouped expenses into the following categories: direct medical, direct non-medical, and indirect. Direct medical expenses included surgery, anesthesia, nursing care, imaging, lab work, and medicine. Direct non-medical expenses were expenses related to transportation to the hospital and food during the stay. Indirect expenses were the lost opportunity costs incurred by the hospitalization. This included lost wages for the household during the hospitalization as well as the transportation, food, and lodging for the caregivers who come to care for a woman while she delivers.

CHE is typically defined either as spending greater than 10% of total annual household expenditure or greater than 40% of annual expenditure, not including subsistence needs [27, 28]. For this paper, we used the 10% of total expenditure definition to align with the Sustainable Development Goal 3.8.2 [29]. We used the World Bank definitions for poverty, defined as a daily expenditure of less than $3.20 in purchasing power parity (PPP) per person per day, and extreme poverty, defined as a daily expenditure below $1.90 per person per day [30, 31]. C-section expenses are thus defined as an impoverishing expenditure if the addition of the c-section expenses pushed the individual below the $3.20 per person per day poverty line or further into poverty below the $1.90 per person per day threshold [32, 33].

### Data analysis

We report demographic data with frequencies or with median and interquartile ranges (IQRs). Medians were used for data involving expenditures and income which are skewed. Patient reported household expenses were summed to obtain the annual total household expenditure. We calculated whether the direct medical costs, all direct costs, or all (direct + indirect) costs met the definition of CHE (i.e. > 10% of the calculated annual total household expenditures). We report the CHE overall and stratified by *Ubudehe* categories.

To determine impoverishing expenditure, we calculated the daily expenditure per person in the household and then subtracted the c-section-related expenses. We report the number and percent of households that were below the poverty thresholds before and after incurring the c-section expenses. For all estimates, we report 95% confidence intervals calculated using the Wilson method. We used the Chi-squared test to compare groups.

In addition to reporting on catastrophic and impoverishing expenditures for this population, we modeled hypothetical scenarios of different insurance strategies. The modeled scenarios were as follows: Scenario 1, assuming there is no insurance coverage for anyone; Scenario 2, 64% of medical costs are covered for everyone (the mean covered proportion in sub-Saharan low-and middle-income countries (LMICs) [34]); Scenario 3, 90% of medical costs are covered for everyone; Scenario 4, 100% coverage of medical costs are covered for all patients; and Scenario 5, 100% coverage of medical costs and ambulance fees are covered for all patients. In each of these scenarios, the modelled population replicates the observed one, and only the above possible insurance strategies are altered. To do this, we first estimated the total cost for services in the absence of insurance coverage by multiplying the medical costs and ambulance fees for all CBHI-paying (*Ubudehe* categories 2–4) patients by 10, since patients in those categories have a 10% copay. Since medical costs were only known for patients covered in *Ubudehe* Categories 2 or 3 (no patients in *Ubudehe* Category 4 were observed in our study, and *Ubudehe* Category 1 patients have 0% copay), we bootstrapped costs from this subgroup to fill in the missing data for patients under other insurance coverage, keeping other variables as collected. We performed 1000 bootstrap replicates and assessed the posterior distributions using diagnostic plots (Supplemental Fig. 1). We did a Monte Carlo analysis on the resulting datasets, replicating the main analysis by calculating the proportion of patients who experienced CHE in each dataset and then reporting the average under each scenario.

For descriptive tables, all tradable expenses (such as medications and travel costs) were converted from RWF to United States Dollars (USD) using the nominal exchange rate at the beginning of the study (854.13), and all non-tradeable expenses (such as salaries) using the purchasing power parity exchange rate. (Rwanda PPP for personal consumption 2017 = 322.21) [35]. Calculations for impoverishing and CHE were performed in RWF. Our results were not sensitive to the choice of exchange rate. Data analysis was performed using R (version 3.5.1, Vienna, Austria).

### Ethical approval

All women were enrolled after providing a written informed consent. Ethical approval for the study was granted by the Rwandan National Ethics Committee (Kigali, Rwanda, No. 848/RNEC/2016) and the Partners Human Research Committee (Boston, USA, No. 2016P001943/MGH).

## Results

In total, 340 patients were interviewed with a median age of 26 years (IQR: 22, 31) (Table 1). Three (0.9%) had HIV, but the rest had no major medical problems. Nearly all (*n* = 330, 97.1%) patients had insurance, the majority (*n* = 310, 91.2%) with CBHI. Median household size was four (IQR: 3, 5) and most women were either married (*n* = 133, 39.2%) or living with their partner (*n* = 173, 51.0%). About 15% (*n* = 50) of the insured study patients were in the lowest *Ubudehe* category. Median travel time from home to the health center was 30 minutes (IQR: 15, 45) and from the health center to the hospital was 4 hours (IQR: 1, 12). Median length of stay in the hospital was 3 days (IQR: 3, 4). Over two-thirds (*n* = 234, 68.8%) of patients were farmers. The summed median annual nominal total household expenditure was $439.01 ($259.04, $610.87), or $1163.99 (IQR: 686.82, 1619.67 using PPP), equating to a nominal median daily expenditure per person of $0.30 (IQR: $0.17, $0.45), or $0.80 (IQR: $0.45, $1.22) using PPP. Nearly all patients (*n* = 334, 98.8%) resided in households that would be classified as poor with 93.5% (*n* = 316) residing in extremely poor households.

**Table 1:** Patient characteristics for c-section patients at KDH (*N* = 340)

	Median (IQR) or n (%)
**Age**	26 (22–31)
**Insurance**	330 (97.1%)
None	10 (2.9%)
Private	20 (5.9%)
CBHI:	310 (91.2%)
*Ubudehe* Category 1	50 (16.1%)
*Ubudehe* Category 2/3	260 (83.9%)
**Travel time to health center** (min)	30 (15–45)
**Travel time to hospital** (min)	240 (60–720)
**Length of stay** (days)	3 (3–4)
**Health conditions (*n*** **= 339)**	
HIV	3 (0.9%)
Obesity	0 (0%)
Diabetes	0 (0%)
Anemia	0 (0%)
**Occupation**	
Farmer	234 (68.8%)
Unskilled labor	43 (12.6%)
Employed	29 (8.5%)
Self-employed	30 (8.8%)
House-wife	4 (1.2%)
**Household size**	4 (3–5)
**Marital status (*n*** **= 339)**	
Single	32 (9.4%)
Married	133 (39.2%)
Living with a partner	173 (51.0%)
Divorced	0 (0.0%)
Widowed	1 (0.3%)
**Reported Annual Income**^a^	$1489.71 ($893.83–$2234.57)
**Expenditures (*n*** **= 338)**	
Calculated annual household expenditure^b^	$439.01 (259.04, 610.87)
Daily expenditure/person, median^b^	$0.30 ($0.17–$0.45)

^a^Converted to USD using 2017 PPP

^b^Converted to USD using 2018 nominal exchange rate

The median medical expenditure paid OOP per patient at time of service was $9.36 (IQR: $7.83, $10.52), with consumables, medications, and the surgical procedure being the costliest expenses (Table 2). Median transportation cost to the health center was $1.17 (IQR: $0, $2.34) and from the health center to the hospital was $1.83 (IQR: $0, $2.81). Two patients took an ambulance from their home to the health center and 235 (67.9%) took one from the health center to the hospital. The median direct cost, including transportation, was $19.29 (IQR: $15.54, $25.22). Finally, when all direct and indirect costs are added together, median cost paid by the patient for c-section was $29.78 (IQR: $22.29, $39.81).

**Table 2:** Summary of out-of-pocket expenditures for c-section hospitalization, direct and indirect costs by paying status (USD)^a^

	Total Median (IQR) (***n*** = 340)	***Ubudehe*** Category 1 Median (IQR) (***n*** = 50)	***Ubudehe*** Category 2/3 Median (IQR) (***n*** = 50)	No Insurance Median (IQR) (***n*** = 10)	Private Insurance Median (IQR) (***n*** = 20)
**Direct Medical**					
Consumables	$2.51 (1.92–3.03)	$0.00	$2.56 (2.20–2.94)	$21.59 (14.49–27.58)	$3.94 (3.23–4.30)
Medications	$2.12 (1.57–2.58)	$0.00	$2.21 (1.82–2.57)	$17.87 (15.05–25.18)	$2.72 (2.52–3.31)
Procedure	$1.91 (1.91–1.91)	$0.00	$1.91 (1.91–1.91)	$66.97 (66.97–66.97)	$7.68 (7.34–7.68)
Labs	$1.18 (0.23–1.18)	$0.00	$1.18 (0.39–1.18)	$27.18 (13.55–40.81)	$4.44 (1.47–4.44)
Consultations	$0.31 (0.27–0.38)	$0.00	$0.31 (0.31–0.35)	$7.69 (7.39–10.18)	$1.09 (0.97–1.20)
Nursing care	$0.27 (0.15–0.35)	$0.00	$0.28 (0.21–0.34)	$9.63 (7.18–10.66)	$1.09 (0.80–1.19)
Imaging	$0.25 (0.00–0.25)	$0.00	$0.25 (0.00–0.25)	$0.00 (0.00–6.42)	$0.85 (0.00–0.92)
Hospitalization	$0.22 (0.11–0.28)	$0.00	$0.22 (0.17–0.28)	$5.82 (3.88–7.27)	$0.70 (0.62–0.90)
**TOTAL**	**$9.36 (7.83–10.52)**	**$0.00**	**$9.53 (8.54–10.38)**	**$158.89 (143.41–173.33)**	**$22.45 (21.28–24.72)**
**Direct Non-Medical**					
Food	$4.68 (2.93–5.85)	$3.51 (2.34–5.85)	$4.10 (2.93–5.85)	$2.34 (2.34–2.93)	$9.60 (5.85–11.71)
Transportation to health center	$1.17 (0.00–2.34)	$1.17 (0.00–1.76)	$1.17 (0.00–2.34)	$1.76 (1.32–1.90)	$0.59 (0.00–0.59)
Transportation to hospital	$1.83 (0.00–2.81)	$0.05 (0.00–2.26)	$1.87 (0.00–2.81)	$18.73 (3.71–28.09)	$0.00 (0.00–2.02)
Caregiver	$2.34 (0.00–5.85)	$1.87 (0.00–5.71)	$2.34 (0.00–5.33)	$0.70 (0.00–1.11)	$6.44 (3.07–11.91)
**TOTAL DIRECT**	**$19.29 (15.54–25.22)**	**$8.08 (5.27–15.22)**	**$20.18 (16.69–25.14)**	**$187.25 (156.36–208.95)**	**$42.55 (29.37–51.16)**
**Indirect**					
Lost wages	$9.31 (6.52–15.52)	$9.31 (6.52–14.90)	$9.31 (6.52–15.52)	$6.52 (6.52–8.61)	$17.07 (0.00–31.04)
**TOTAL WITH INDIRECT**	**$29.78 (22.29–39.81)**	**$16.33 (14.13–25.38)**	**$30.47 (24.66–39.16)**	**$201.94 (177.54–221.37)**	**$55.44 (44.74–69.78)**

^a^Converted to USD using nominal exchange rate

**Figure 1:**
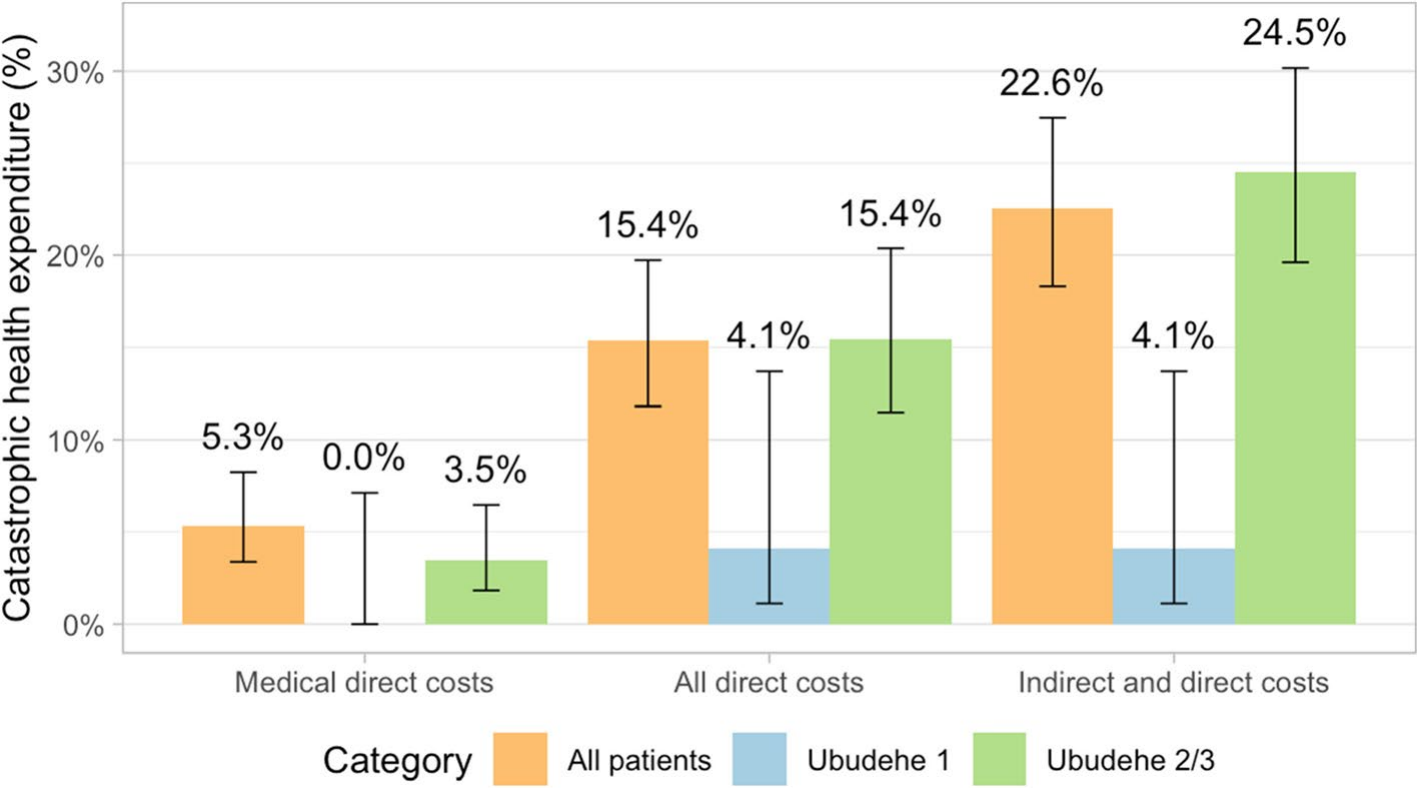
**Fig. 1 **Rates of CHE by CBHI category

Figure 1 shows substantially higher rates of CHE for all patients and by *Ubudehe* category when adding direct and indirect costs to the medical costs alone. When only considering medical costs paid to the hospital, the risk of CHE is 5.3%; but when direct costs are added, this rises to 15.4% (*p* < 0.001), and with indirect costs, 22.6% of households experienced CHE (p < 0.001). The risk of CHE is higher for patients in the *Ubudehe* 2 and 3 categories than for patients in the *Ubudehe* 1 category (*p* = 0.0026). Before the hospitalization, 98.8% of patients were poor and 93.5% were extremely poor by the World Bank definitions. After hospitalization, these numbers rose to 99.4 and 96.7% respectively. Nearly half (*n* = 164, 48.2%) of patients borrowed money to pay for OOP costs related to the c-section with a median amount borrowed of $16.39 (IQR: $10.53, $23.41). Paying patients in *Ubudehe* categories 2 and 3 were more likely to have to borrow money to pay for care than patients with other insurance (*p* < 0.001), but at least one third of patients in all groups had to borrow money (Fig. 1). 43 patients (12.7%) had to sell possessions and one lost a job due to the surgery. Six patients paid for a caregiver during the hospitalization.

**Figure 2:**
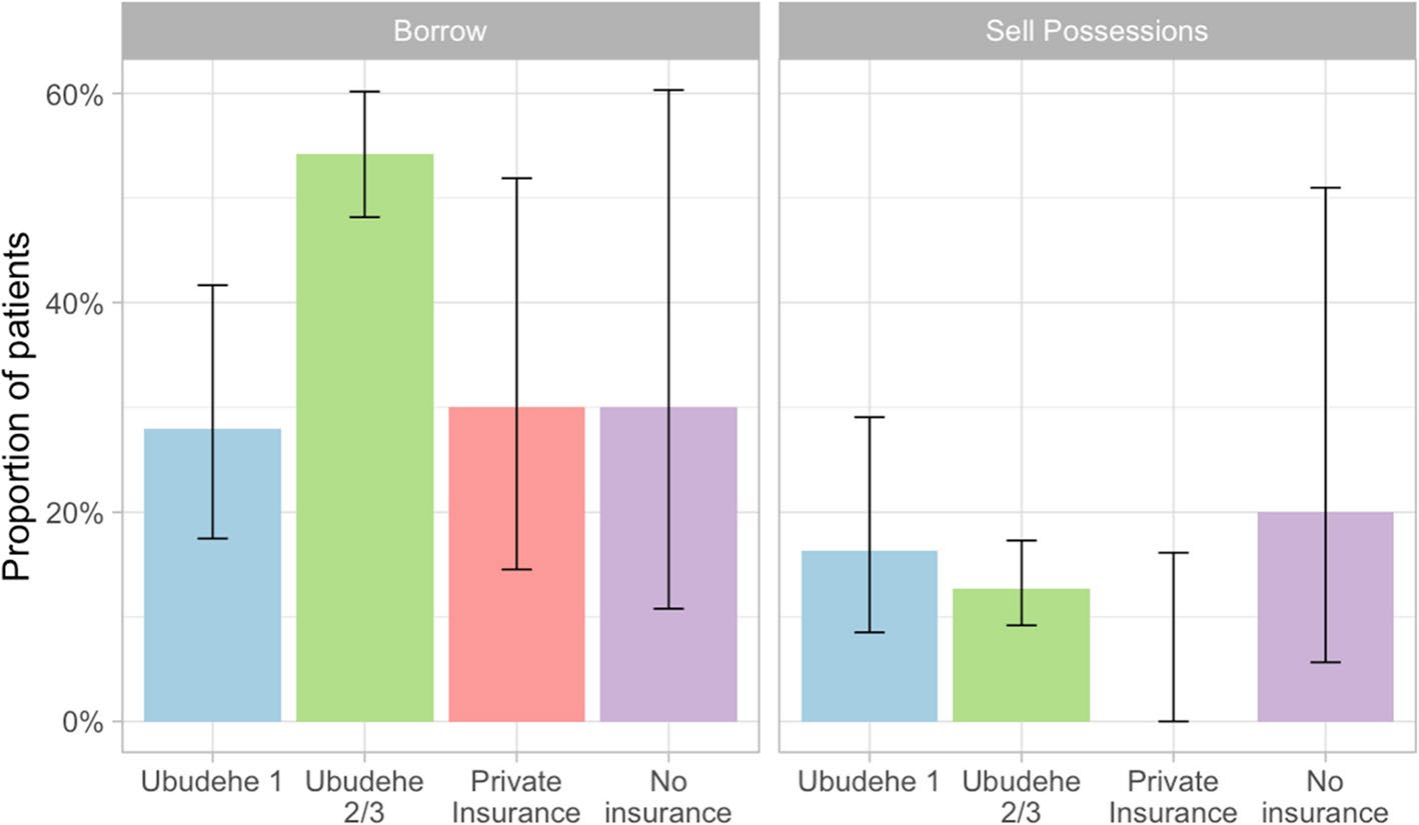
**Fig. 2 **Proportion of patients who borrowed money or sold possessions to pay for c-section expenses by insurance type

Under the assumptions of the insurance scheme modeling, CBHI dramatically reduces the rates of CHE. If there was no insurance coverage, 88% of households would experience CHE paying for medical costs alone and 95% if all direct costs are included (Fig. 1). At 64% coverage, the rates of CHE would be 35 and 56% for medical and all direct costs respectively. For 90% coverage, the CHE rates are 4 and 17% respectively. Finally, the model shows that even if all medical costs and transportation costs were covered, 3–4% of patients would still experience CHE from indirect costs.

**Figure 3:**
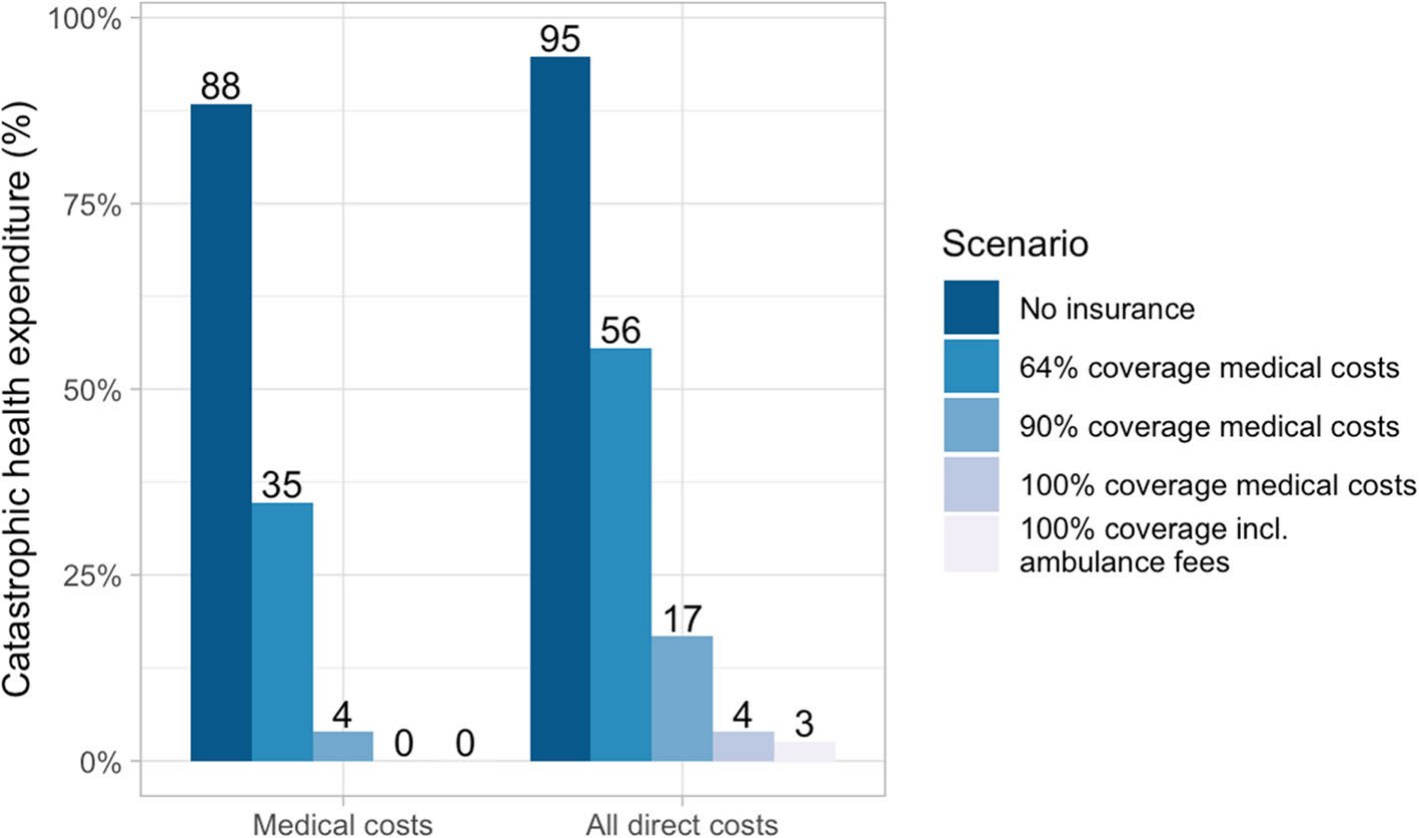
**Fig. 3 **Rates of CHE by insurance coverage models

## Discussion

Despite nearly universal access to CBHI in Rwanda, up to a quarter of patients in our study still experienced CHE when paying for the direct and indirect costs of this essential surgical care. Eliminating the medical cost of care is not sufficient to alleviate this burden as it is the addition of transportation, food, and other indirect costs that cause financial catastrophe for many patients. Even modest nonmedical direct and indirect costs such as the median of $30 for a c-section are nontrivial for poor patients. Additional informal payments are noted in some African contexts [22], and these would certainly increase this OOP expenditure and rates of CHE. However, given strongly enforced anti-corruption policies throughout Rwanda, hospital leadership felt that such payments are rare, and hence this was not assessed in our study. There are, however, other nonmonetary costs even when patients are able to find the finances for surgical care: nearly half of patients had to borrow money from family or friends to pay their bill, thereby incurring an informal debt they would have to repay [36], and 12% had to sell possessions.

Though we found disappointingly high rates of CHE, these rates would be exponentially higher if there was no insurance at all. Our model showed that those with subsidized care had lower rates of CHE. Even if medical costs and transportation are fully subsidized, there will still be a low rate of CHE (3–4%) for impoverished patients. Not surprisingly, though, we found that when a higher percentage of costs are covered by insurance or by the government, far fewer patients are in danger of CHE. Consequentially, in the current system, the poorest patients who are fully subsidized with free care are better protected, while those in the next tier are more vulnerable to financial catastrophe. Notably, for all groups, c-section is not categorized as *impoverishing*. This is largely because across the *Ubudehe* categories, these women and their families are already classified as poor and most are extremely poor, hence are not considered impoverished by the expense according to the definitions used. Despite the introduction of *CBHI,* which reduced rates of OOPs and CHE, inequalities in the population have not been reduced [37]. Thus, further strategies are needed to target the challenges to accessing care for those living in poverty. Informal insurance structures have been shown to be of particular utility in reducing CHE in SSA [38]. In Ghana and Ethiopia, rates of CHE are also much higher in uninsured patients, and CBHI in particular was found to decrease rates of CHE by up to 23.2% [39, 40]. However, studies have also shown inequalities in who chooses to access CBHI with the rich utilizing it more and dropout rates being highest among those required to pay a premium for services [41]. This dynamic has also been anecdotally reported in Rwanda where patients may feel they are being taxed by the compulsory enrollment. In this scenario, voluntary enrollment with more community-level governance could be benficial [11].

Our findings suggest that there is need for greater financial protection for impoverished households in order to achieve the best medical and social outcomes for patients requiring hospital services and surgical care. In the Democratic Republic of Congo, 16% of women experienced CHE related to obstetric and neonatal care, particularly if there were any complications, while in Ghana, substantial rates of CHE were found due to transportation and indirect costs, particularly among patients with complications, despite free maternal health care [42, 43]. These issues extend beyond maternal health and c-sections, with studies from Malawi and Uganda demonstrating high rates of CHE for other surgical conditions despite free surgeries or no user fees [44–47]. A study by Mercy Ships (which provides free surgery to patients) demonstrated that paying for transportation decreased the no-show rate for surgery by 45% [48]. Policy makers could consider such initiatives as well as others that either offset the cost of transportation or decrease the need for transportation for these patients. Strategies to avoid compounding the financial risk in the post-operative period while obtaining the best outcomes might include vouchers to offset the cost of post-operative follow-up, home-based follow-up utilizing community health workers (CHWs), and empowering CHWs with mobile applications on smartphones to facilitate effective detection of surgical site infection and other post-operative complications, thus reducing the demand for in-person post-operative follow up [23].

Importantly, we note that true rates of CHE may be underestimated if patients choose not to have surgery due to associated costs. This is less likely for c-section patients in Rwanda, for whom surgery is usually urgent or emergent. However, when extrapolating to other types of operations including those considered elective initially or definitively, some patients may lack access to care based on financial capacity to pay. Poverty in Rwanda is correlated with lower health care services coverage [49], which suggests that despite financial protection for essential services, overall access to medical care for uncovered services is still threatened. The rate of CHE reported here is likely an underestimate as we did not include post-discharge costs or the cost of care for complications. Our group, in a previous study, found that the cost of travel from home-to-health center was a significant predictor of surgical site infection^50^, potentially suggesting that these expenses are prohibiting the necessary follow-up care.

### Limitations

This study had several key limitations. First, it was only conducted at a single site, which may have local geographic features, though culturally and socioeconomically the population does resemble other parts of rural Rwanda in terms of care seeking behavior and resources. Rwanda also has a unique political context, particularly relating to the CBHI program, and so the results and ensuing policy recommendations may not fully generalize to other LMICs. Second, this study may not capture any patients who did not seek hospital care due to inability to pay for services. That number is likely very small based on the high penetration of CBHI membership in the community around Kirehe. Furthermore, this study only looks at the rates of CHE for one essential procedure which is of value to an entire household, therefore the rates may not represent the financial risk of other surgical procedures which may be considered elective by either the family, the medical community, or the government. Another limitation of our study is that we focused exclusively on the expenses incurred peripartum – from onset of labor up through discharge. These expenses should be considered in addition to any prenatal care expenses as well as postpartum care expenses, particularly for those who experienced complications after discharge. This study may thus underestimate the financial burden of c-section. A subsequent study to capture the expenses and CHE for women delivering via c-section through 30 days post-delivery is underway to better understand total financial risk for this population.

Another known limitation of this type of data collection is that the calculated household expenditure depends on patient memory of their regular expenses and therefore suffers from recall bias. Interestingly, when patients were asked their total monthly expenditure, the amount was generally higher than the sum of its components. Furthermore, in a rural population, households may not have regular expenses but rather have occasional larger purchases related to agriculture or home maintenance. Therefore, estimating daily expenditure may not be a consistent measure of a patient’s true resources. Finally, indirect costs are likely underestimated since lost wages also include recovery time at home post-operatively.

## Conclusions

In conclusion, OOP expenditure for essential surgery confers significant financial risk on already impoverished households, even where government acts in support of promoting UHC via initiatives such as financial subsidization through CBHI. Specifically, this study found that transportation costs to the hospital were a significant burden to families who needed transfer to the hospital for a c-section.. Providing solutions to this, such as providing free or discounted transport for peripartum women in rural areas, could be an area of focus. Additionally, we recognize that the merit of the impoverishment thresholds set by the international community are limited in utility as most of the patients in our study were already below the poverty threshold even prior to emergency surgery. Given that the cost of surgery is already low, rather than reducing cost, consideration ought to be given to ways of decreasing poverty rates in rural Rwanda and increasing the subsidies for those in *Ubudehe* groups 2 and 3 who are most affected. Our study highlights issues around surgical hospitalization bills-driven financial impoverishment and insurance effects for an extremely poor population. Policy makers need to complement the merits of CBHI by devising strategies that address more complex barriers to care that women face when delivering by c-section. This will ensure mitigation of the risk of financial catastrophe while optimizing good health outcomes for mothers and babies at the time of delivery.

## Supplementary Information

**Additional file 1.** 

**Additional file 2.** 

## Data Availability

The datasets used during the current study are available from the corresponding author on reasonable request.

## Abbreviations

CHE: Catastrophic health expenditure
c-section: Cesarean section
CBHI: Community-based health insurance
CHW: Community health worker
IQR: Interquartile range
KDH: Kirehe District Hospital
LMIC: Low and middle income country
NGO: Non-governmental organization
OOP: Out of pocket
POD: Postoperative day
PGSSC: Program in Global Surgery and Social Change
PPP: Purchasing power parity
RWF: Rwandan Francs
SSA: Sub-Saharan Africa
UHC: Universal health coverage
USD: United States Dollar
WHO: World Health Organization
