# Supplementary material for: Does community-based health insurance protect women from financial catastrophe after cesarean section? A prospective study from a rural hospital in Rwanda
Source: BMC Health Serv Res. 2022 May 31;22:717. doi: 10.1186/s12913-022-08101-3 (PMC9153099; doi:10.1186/s12913-022-08101-3)
Supplement: Supplementary file 1 — Additional file 1. [file 12913_2022_8101_MOESM1_ESM.pdf]

Financial Risk Protection Survery

Patient study identification number ________________________________

What is the size of your household, including yourself and new infant? (how many members normally live in your house) ________________________________

What occupation is held by the person who makes the most money in your household (primary breadwinner)?
- ◯ Student
- ◯ Farmer
- ◯ Employed (Government / NGO / Private company)
- ◯ Self-employed
- ◯ House-wife
- ◯ Other
- ◯ Unskilled labor (non-agricultural)

If other, what? ________________________________

How much does your household earn in an average month? ________________________________

How much money does your household spend on food (including oil, salt, sugar, cooking petrol) and water, juice or soda to drink per month? ________________________________

How much money does your household spend on livestock (cows, pigs, goats, chickens etc.) every month? [if not known please indicate yearly expenditure] ________________________________

How much money does your household spend on transport per month? ________________________________

How much does your household pay for your house/apartment in rent or mortgage or housing fees per month (if applicable)? ________________________________

How much money does your household spend on other household items such as clothes, improvements to your house etc per month? [if not known, estimate per year] ________________________________

How much money does your household spend on education per term for all the children you support, including school fees and books? ________________________________

Do you have other regular expenditures?
- ◯ Yes
- ◯ No

If so what are they and how often do you pay them? ________________________________

How much are these other expenditures? ________________________________

Excluding this hospital course, how much money does your household usually spend on health care, including medicines, fees for doctors or hospital visits, fees for traditional healers per year? ________________________________

Hospitalization Associated Expenditures

How many days were you in the hospital?

- ◯ 1
- ◯ 2
- ◯ 3
- ◯ 4
- ◯ 5
- ◯ 6
- ◯ 7 or more

How much did you pay each day you were in the hospital?

____

How much money in total did your household spend for (all)/the attendant(s) to come to the hospital to bring food or look after the patient?

____

Did you have to pay any fees or make any informal payments directly to healthcare workers or hospital employees for your surgery or hospitalization?

- ◯ Yes
- ◯ No

If yes, how much did you pay?

____

Did your household pay anyone else to act as an attendant and take care of the patient during this hospitalization?

- ◯ Yes
- ◯ No

Did you household have to borrow money to pay for this hospitalization?

- ◯ Yes
- ◯ No

If yes, how much?

____

Did your household have to sell land or possessions (including livestock) to pay for this hospitalization?

- ◯ Yes
- ◯ No

Did your household have to permanently stop sending children to school, or did you pay reduced school fees in order to pay for this hospitalization?

- ◯ Yes
- ◯ No

What amount did your household spend on food during the hospitalization?

____

What amount in wages has your household lost due to this hospitalization? (ie. calculate based on how many people have missed work and for how many days and their daily wage)

____

Did you or anyone in your household permanently lose a job as a result of this hospitalization?

- ◯ Yes
- ◯ No

How much did you receive in outside funds from charity or from the social worker/hospital fund to pay for your hospitalization for all goods and services?

____

Hospital Expenses

Hospital electronic ID __________________________________

How much money did the patient spend on this hospitalization? __________________________________

How much money did you spend on medications & other consumables? (from hospital records) __________________________________

How much money did your household spend on laboratory tests? (from hospital records) __________________________________

How much money did you spend on procedures, bandages and dressing supplies? (from hospital records) __________________________________

How much money did your household spend on imaging and x-ray? (from hospital records) __________________________________

How much money did your household spend on any other medical supplies? (from hospital records) __________________________________

How much did you spend on ambulance fees? __________________________________

Did you have to pay any other fees to the hospital besides those we have already mentioned?

- ◯ Yes
- ◯ No

If yes, how much? __________________________________
