# Supplementary figures and images for: Does community-based health insurance protect women from financial catastrophe after cesarean section? A prospective study from a rural hospital in Rwanda
Source: BMC Health Serv Res. 2022 May 31;22:717. doi: 10.1186/s12913-022-08101-3 (PMC9153099; doi:10.1186/s12913-022-08101-3)

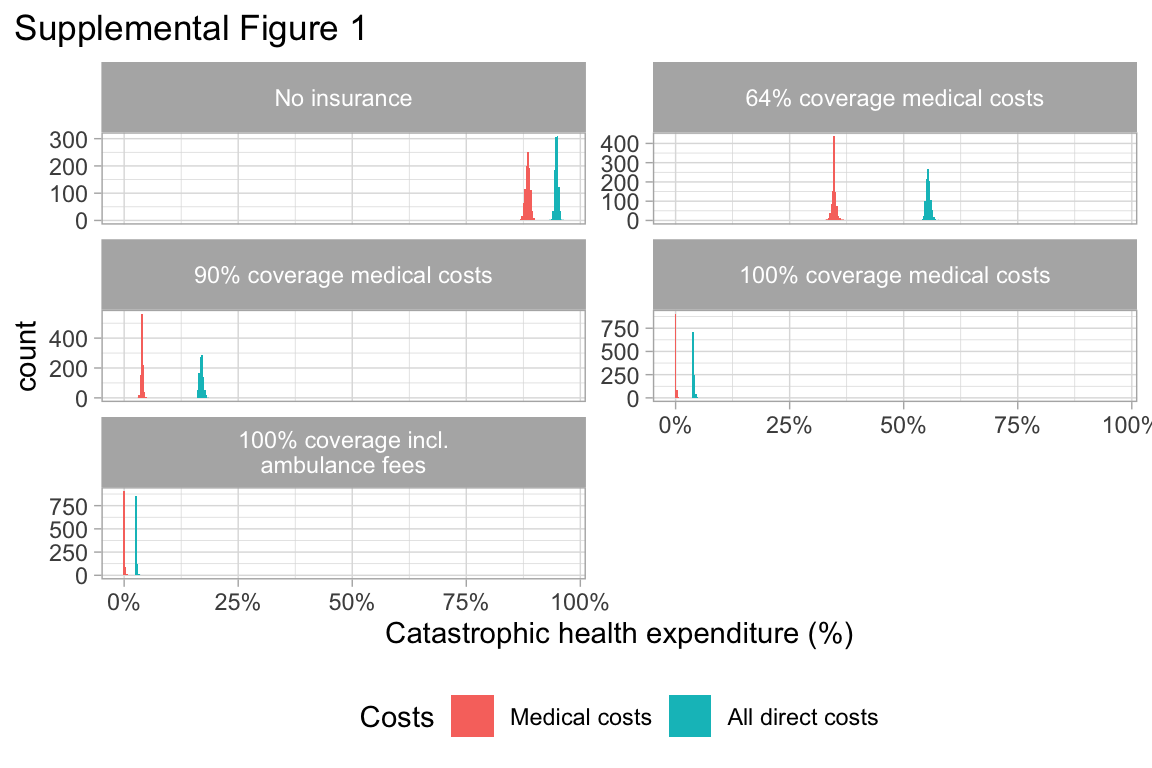

Supplement: Supplementary file 2 — Additional file 2. [file 12913_2022_8101_MOESM2_ESM.docx]
